# Clinical features of children with anti-CFH autoantibody-associated hemolytic uremic syndrome: a report of 8 cases

**DOI:** 10.1080/0886022X.2022.2089167

**Published:** 2022-06-22

**Authors:** Qian Li, Xinxin Kong, Minle Tian, Jing Wang, Zhenle Yang, Lichun Yu, Suwen Liu, Cong Wang, Xiaoyuan Wang, Shuzhen Sun

**Affiliations:** a Department of Pediatric Nephrology and Rheumatism and Immunology, Shandong Provincial Hospital, Cheeloo College of Medicine, Shandong University, Jinan, P. R. China; b Department of Pediatric Nephrology and Rheumatism and Immunology, Shandong Provincial Hospital, Affiliated to Shandong First Medical University, Jinan, P. R. China; c School of Basic Medical Sciences, Shandong First Medical University, Taian, Shandong, China

**Keywords:** Hemolytic uremic syndrome, anti-CFH autoantibody, children, eculizumab, cyclophosphamide, mycophenolate mofetil

## Abstract

**Objective:**

To explore the clinical characteristics, treatment protocol and prognosis of children with anti-complement factor H (CFH) autoantibody (Ab)-associated hemolytic uremic syndrome (HUS).

**Methods:**

Clinical data of 8 patients with anti-CFH Ab-associated HUS who were admitted to Shandong Provincial Hospital from January 2011 to December 2020 were collected retrospectively.

**Results:**

The age at disease onset ranged between 5.83 and 13.5 years, with a male: female ratio of 1.67:1. The time of onset was distributed from May to June and November to December. Digestive and upper respiratory tract infections were common prodromal infections. Positivity for anti-CFH Ab and reduced C3 levels were observed among all patients. Heterozygous mutation of the CHFR5 gene (c.669del A) and homozygous loss of the CFHR1 gene [loss2(EXON:2-6)] were found in two patients. All patients received early treatment with plasma exchange and corticosteroid therapy. Six patients were given immunosuppressive agents (cyclophosphamide and/or mycophenolate mofetil) for persistent proteinuria. The follow-up period was 12–114 months. Four of 8 patients achieved complete remission, 3 achieved partial remission, and 1 died. Relapse occurred in two patients.

**Conclusion:**

Children with anti-CFH Ab-associated HUS were mainly school-aged and predominantly male, with onset times of summer and winter. Digestive and upper respiratory tract infections were common prodromal infections. Plasma exchange combined with methylprednisolone pulse therapy in the acute phase and cyclophosphamide or mycophenolate mofetil treatment for maintenance can be utilized in children with anti-CFH Ab-associated HUS if eculizumab is not available.

## Introduction

Hemolytic uremic syndrome (HUS) is a thrombotic microangiopathy (TMA) with three major clinical features: microvascular hemolytic anemia, acute kidney injury, and thrombocytopenia. HUS is divided into infection-related HUS, secondary HUS, and atypical HUS (aHUS) [1,2]. aHUS is mainly caused by abnormal complement replacement pathways, including hereditary and acquired complement functional defects. Complement factor H (CFH) is the main fluid phase regulator of the complement alternative pathway, and inherited defects and autoantibody formation in CFH are the two main pathogenic mechanisms that cause aHUS. Acquired complement functional defects mainly refer to anti-CFH autoantibody (Ab)-associated HUS. Anti-CFH Ab-associated HUS has serious clinical manifestations, with a mortality rate of up to 25%, and 20–29% of survivors progress to kidney failure; the recurrence rate of anti-CFH Ab-associated HUS is approximately 20–25% [3]. At present, immunosuppressive therapy is recognized internationally as an effective treatment [4], but there is no uniform standard for the selection and treatment protocol of immunosuppressive agents.

Herein, we collected the clinical data of 8 cases of anti-CFH Ab-associated HUS in our hospital from 2011 to 2020 and retrospectively analyzed the clinical characteristics, treatment protocols and prognosis of these pediatric patients.

## Materials and methods

### Patients

Clinical data of 8 patients with anti-CFH Ab-associated HUS who were admitted to Shandong Provincial Hospital from January 2011 to December 2020 were collected retrospectively. All patients were treated according to the same protocol. The diagnosis of HUS was based on the following criteria: acute hemolytic anemia, fragmented erythrocytes in a peripheral blood smear, thrombocytopenia (platelet count <150 × 109/L), elevated levels of lactate dehydrogenase (LDH), negative Coombs’ test, and/or acute kidney injury [5].

The inclusion criteria were as follows: (1) <14 years of age; (2) clinically consistent diagnostic criteria for aHUS [6]; and (3) positive serum anti-CFH Ab. The exclusion criteria were disseminated intravascular coagulation (DIC) caused by other reasons, immune hemolytic anemia, Shiga toxin-producing Escherichia coli HUS (STEC-HUS), secondary HUS, and anti-CFH antibody-negative thrombotic thrombocytopenic purpura (TTP) (ADAMTS13 activity <5%).

The current study was approved by the ethics committee of our hospital with the approval number NO.2022-253, and all patients’ legal guardians signed written informed consent.

### Data collection

Clinical and pathological data were collected, mainly consisting of general indexes (including sex, age and time of onset), symptoms of prodromal infections, clinical manifestations, laboratory tests (including hemoglobin, platelets, urea nitrogen, serum creatinine, 24 h urinary protein content, complement C3, C4, CFH and anti-CFH autoantibodies, etc.), kidney biopsy, genetic testing, treatment and prognosis.

### Detection of serum CFH levels

Serum CFH levels were detected by ELISA [5]. All assays were run in duplicate, and when standard errors were >10%, samples were routinely reanalyzed. Serial concentrations of commercially available highly purified human factor H were used to develop a standard curve. The linear portion of the curve was subsequently used for the measurement of serum factor H.

### Detection of plasma anti-CFH autoantibodies

Plasma anti-CFH immunoglobulin G (IgG) titers were detected by ELISA [5]. Each sample was tested in duplicate. Anti-CFH IgG titers were expressed as arbitrary units per milliliter (AU/ml) and were calculated as (S − B) × 1000/(P − B), where S was the optical density of the test sample diluted at a ratio of 1:100, B was the absorbance of the blank, and P was the absorbance of the reference positive plasma diluted to a ratio of 1:100, which was assigned an arbitrary titer of 1000 AU/ml. The cutoff value was the mean titer + 2 × standard deviation (SD) of 100 individual healthy controls. Titers above the cutoff value were considered positive.

### Kidney histopathology

Kidney biopsy was performed on 3 of 8 patients. A spring-loaded needle biopsy kit was used to obtain kidney tissue samples under the real-time guidance of a B-ultrasonic detection system. After percutaneous kidney puncture biopsy, the kidney tissue was stained [hematoxylin-eosin (HE) staining, periodic acid-Schiff (PAS) staining, hexamine silver (PASM) staining and Masson staining], and electron microscopy and immunofluorescence were performed (IgA, IgG, IgM, Fib, C1q, C3).

### Gene testing

Whole-exome sequencing (WES) was performed on 5 of 8 patients. Whole blood samples (2–5 mL) from the affected children and their parents were collected. DNA extraction was carried out, and the whole exome was captured and sequenced with the IDT xGen exome research panel v2.0 full exon capture chip. The variation classification adopts the three-factor classification system and the ACMG (American Medical Genetic Society) gene variation classification system. For suspected pathogenic mutations, Sanger sequencing was performed with an ABI3730 sequencer after PCR of the target sequence, and the verification results were obtained with sequence analysis software.

### Treatment response

The response to therapy included complete remission, partial remission, and treatment failure [5]. Complete remission was defined by normalization of hematologic parameters [including hemoglobin (Hb) >100 g/L, platelets (Plt) >150 × 109/L, lactate dehydrogenase (LDH) <450 U/L] and kidney function [serum creatinine (SCR) <80 μmol/L, no proteinuria]. Partial remission was defined by incomplete normalization of laboratory parameters or proteinuria. Treatment failure was defined by death or chronic kidney disease (CKD).

### Statistical analysis

The statistical software SPSS version 18.0 (IBM Corp., Armonk, NY, USA) was used for the statistical analyses. The quantitative data are expressed as the median with interquartile range (IQR).

## Results

### General data

There were 5 males and 3 females, with a male:female ratio of 1.67:1. The age at disease onset ranged between 5.83 and 13.5 years (median 6.67 years, 6.02–9.03 years). The time of onset was distributed from May to June and November to December. All patients had no other kidney diseases or family histories of thrombotic microangiopathy.

### Symptoms of prodromal infection

Prodromal infection symptoms were present in 87.5% (7/8) of patients, of whom 75% (6/8) had digestive symptoms such as vomiting and abdominal pain, 62.5% (5/8) had symptoms of upper respiratory tract infection such as fever and cough, and 50% (4/8) had coinfection of the respiratory system and digestive system. The symptoms of the digestive system mainly included nausea, vomiting, abdominal pain or diarrhea without blood stools.

### Clinical manifestations

All patients showed typical manifestations of HUS, such as hemolytic anemia, thrombocytopenia, and acute kidney injury. All patients showed edema, gross hematuria, foamy urine, and jaundice. Seven patients (87.5%) presented with bleeding points on the skin and mucous membranes. Additionally, gastrointestinal bleeding and/or nosebleed was present in 2 patients (25%). Oliguria or anuria was seen in 6 cases (75%). Five patients (62.5%) presented with liver involvement. Hypertension was present in 2 patients (25%). One patient (25%) presented with pancreatitis (Case 4). One patient (25%) died of pulmonary hemorrhage (Case 1). One patient (25%) presented with convulsions caused by hypertension encephalopathy (Case 8) (Table 1).

**Table 1.** Clinical features of 8 children with anti-CFH Ab-associated HUS.

	Case 1	Case 2	Case 3	Case 4	Case 5	Case 6	Case 7	Case 8
Age (years)	5.83	7	7.33	6.33	9.59	13.5	5.92	6.33
Sex	Male	Male	Male	Male	Female	Male	Female	Female
Prodrome	Vomiting	Vomiting, abdominal pain, fever, cough	Vomiting, fever, cough	Vomiting, abdominal pain, diarrhea	Vomiting, cough	Cough	None	Vomiting, abdominal pain, fever, cough
Time of onset	November	May	December	November	May	November	June	November
Jaundice	Yes	Yes	Yes	Yes	Yes	Yes	Yes	No
Petechia	Yes	Yes	Yes	Yes	Yes	Yes	Yes	No
Hypertension	No	No	No	Yes	No	No	No	Yes
Oliguria/Anuria	Yes	Yes	No	Yes	No	Yes	Yes	Yes
Edema	Yes	Yes	Yes	Yes	Yes	Yes	Yes	Yes
Hematuria	Yes	Yes	Yes	Yes	Yes	Yes	Yes	Yes
Proteinuria	Yes	Yes	Yes	Yes	Yes	Yes	Yes	Yes
Hepatic involvement	No	Yes	Yes	Yes	No	Yes	Yes	No
Other extrarenal involvement	Pulmonary hemorrhage, gastrointestinal hemorrhage, multiple organ failure	no	Epistaxis, gastrointestinal hemorrhage	Pancreatitis	No	No	No	Convulsions, hypertensive encephalopathy, severe pneumonia, respiratory failure

### Laboratory examination

**Figure 1.**
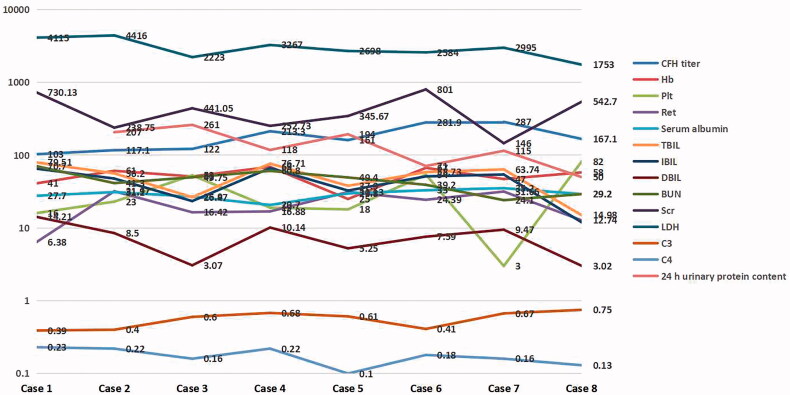
The laboratory results of 8 cases with anti CFH Ab associated HUS.

In the acute phase, all patients tested positive for anti-CFH autoantibodies. The titers of CFH were normal in 2 patients (25%) and reduced in 6 patients (75%). All patients presented with moderate to severe anemia and thrombocytopenia, high levels of lactate dehydrogenase (LDH), a sharply increased ratio of reticulocytes, and severe acute kidney injury, with elevated levels of serum creatinine and urea nitrogen. Hypoalbuminemia and hyperbilirubinemia (mainly indirect bilirubin elevation) were present in 7 of 8 patients (87.5%). The serum levels of C3 were reduced, and serum C4 levels were normal in all 8 patients. The urinary protein quantification for all 24 h showed that all patients presented with gross proteinuria (Table 2, Figure 1).

**Table 2.** Laboratory results of 8 children with anti-CFH Ab-associated HUS.

	Case 1	Case 2	Case 3	Case 4	Case 5	Case 6	Case 7	Case 8
Anti-CFH IgG	Positive	Positive	Positive	Positive	Positive	Positive	Positive	Positive
CFH titer (µg/ml)	103 ↓	117.1 ↓	122 ↓	213.3 ↓	161 ↓	281.9	287	167.1 ↓
Hb (g/L)	41	61	51	69	25	67	47	58
Plt (x10^9/L)	16	23	53	19	18	54	3	82
Ret (%)	6.38	31.97	16.42	16.88	31.13	24.39	31.66	12.74
Serum albumin (g/L)	27.7	31.1	26.9	20.7	29.8	33	35.2	29.2
TBIL (μmol/L)	79.51	56.2	26.47	76.71	37.9	58.73	63.74	14.98
IBIL (μmol/L)	65.3	47.7	23.4	66.57	32.65	51.14	54.27	11.96
DBIL (μmol/L)	14.21	8.5	3.07	10.14	5.25	7.59	9.47	3.02
BUN (mmol/L)	70.7	41.3	49.75	60.8	49.4	39.2	24.1	29.2
Scr (μmol/L)	730.13	238.75	441.05	252.73	345.67	801	146	542.7
LDH (U/L)	4115	4416	2223	3267	2698	2584	2995	1753
C3 (g/L)	0.39	0.4	0.6	0.68	0.61	0.41	0.67	0.75
C4 (g/L)	0.23	0.22	0.16	0.22	0.1	0.18	0.16	0.13
24 h Urinary protein content (mg/kg)	–	207	261	118	194	71	115	50
Renal biopsy	No	No	Yes	Yes	No	Yes	No	No
Gene mutation	–	–	No definite pathogenic mutation	–	No definite pathogenic mutation	No definite pathogenic mutation	CFH R5 heterozygous mutation	CFHR1 homozygous loss

CFH: complement factor H; Hb: hemoglobin; Plt: platelet; Ret: reticulocyte; TBIL: total bilirubin; IBIL: indirect bilirubin; DBIL: direct bilirubin; BUN: blood urea nitrogen; Scr: serum creatinine; LDH: lactate dehydrogenase; C3: complement 3; C4: complement 4.

**Figure 2.**
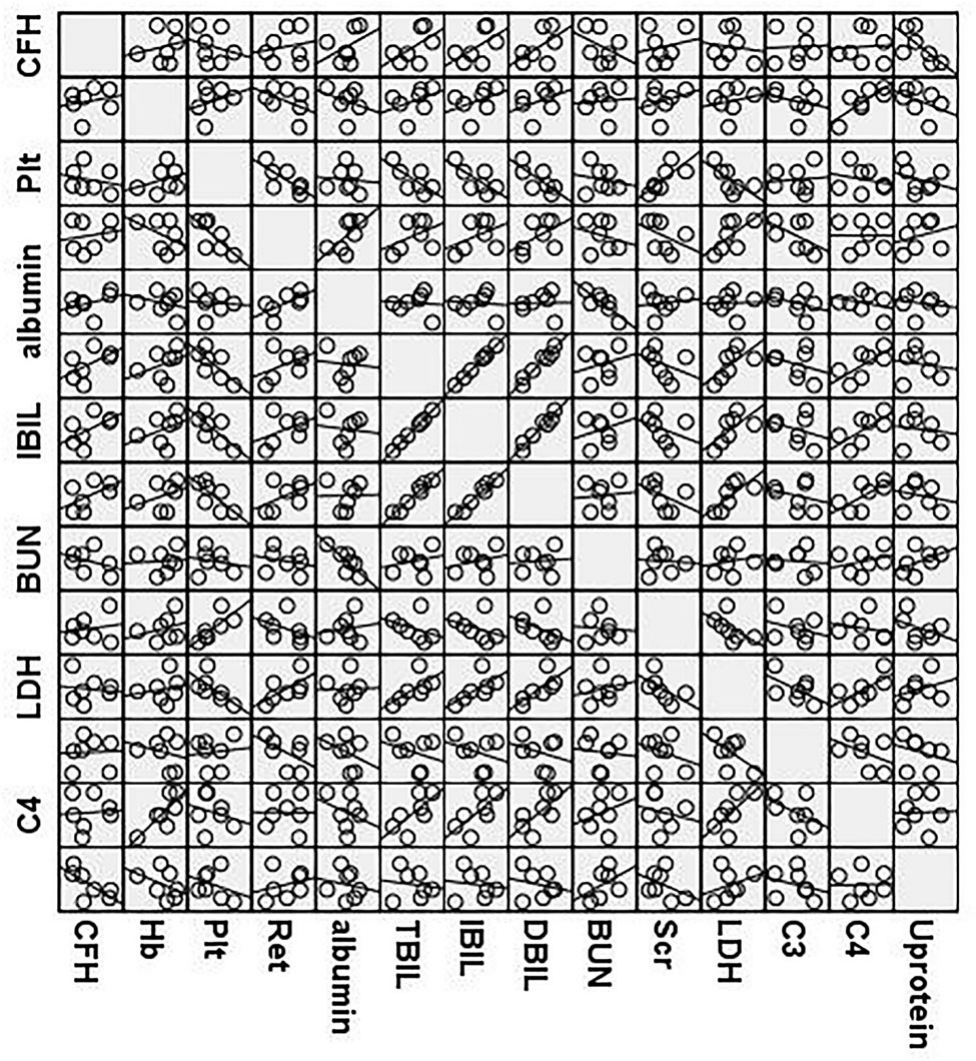
A correlation matrix scatter plot between CFH titer and other laboratory parameters of 8 cases with anti CFH Ab associated HUS.

A correlation matrix between CFH titer and regular parameters ( hemoglobin, platelets, ratio of reticulocytes, albumin, total bilirubin, indirect bilirubin, direct bilirubin, urea nitrogen, serum creatinine, LDH, complement C3, C4, 24 h urinary protein content ) has been made. However, no significant correlation between CFH titer and the above parameters was observed (Figure 2).

### Kidney pathological features

Three of 8 patients underwent kidney biopsy (Table 2). Under a light microscope, mild to moderate segmental proliferation of the mesangial region and shrinkage of the basement membrane were observed. Glomerular capillary tuft collapse was observed in 1 case. Moreover, segmental glomerulosclerosis was observed in 1 or 2 locations in 2 patients. Two or 3 balloon adhesions were found in 2 patients. Swollen arteriolar endothelial cells were observed in 1 patient, and microthrombi were found in 1 patient. Direct immunofluorescence analysis revealed that one patient had positive staining for IgM (2+) and F (2+), one patient had positive staining for IgM (3+), and one patient had no immune complex deposition. In addition, the immune complexes were distributed in granular form along the capillary wall. Under an electron microscope, swollen endothelial cells of glomerular capillaries, widened subendothelial space, and fused foot process cells were observed. One patient appeared to have cellulose-like substances deposited under the endothelium.

### Genetic testing

Five of the 8 patients underwent genetic testing (Table 2). Heterozygous mutation of the CHFR5 gene (c.669del A) and homozygous loss of the CFHR1 gene [loss2 (EXON:2-6)] were found in two patients (Cases 7 and 8), and no disease-related mutations were found in the other three patients.

### Treatment

All patients received early treatment with plasma exchange and corticosteroid therapy. One patient (case 7) received treatment with plasma exchange and 2 courses of methylprednisolone pulse (MPP) therapy. One patient (case 8) received treatment with plasma exchange 9 times, hemodialysis and continuous kidney replacement therapy (CKRT) therapy with MPP therapy (1 d, stopped for severe infection). Five patients received treatment with plasma exchange 5–7 times at first, and 1–3 courses of MPP were adopted later due to poor control of hemolysis. One patient (case 1) received treatment with plasma infusion and plasma exchange and two rounds of high-dose MPP on the 4th day after admission. However, he unfortunately died of pulmonary hemorrhage on the 9th day. The remaining 7 patients continued to receive oral corticosteroid (prednisone) treatment. Six patients were given immunosuppressive agents for persistent proteinuria. Among them, 3 patients received cyclophosphamide (CTX) pulse therapy after the acute phase. The other 3 patients were treated with mycophenolate mofetil (MMF) within six months after the acute phase. Two patients received MMF after CTX therapy for persistent proteinuria and relapse after 14 months (Table 3).

**Table 3.** Treatment and prognosis of 8 children with anti-CFH Ab-associated HUS.

	Case 1	Case 2	Case 3	Case 4	Case 5	Case 6	Case 7	Case 8
Treatment								
Plasma infusion	Yes	No	No	No	No	No	No	No
Plasma exchanges (times)	1	2	5	9	8	5	7	9
CKRT/hemodialysis	Yes	Yes	No	Yes	No	Yes	No	Yes
Steroids	MPP	MPP	MPP	MPP	MPP	MPP	MPP	MPP
Immunosuppressants	none	MMF	CTX, MMF	CTX	MMF	MMF	CTX, MMF	CTX
Time of follow-up (months)	–	114	70	57	65	72	62	12
Prognosis	death	CR	PR	CR	CR	CR	PR	PR
Time to recovery of blood parameters and renal function (days)	–	13	29	24	41	23	25	–
Time to recovery of urine protein (months)	–	20	–	3	19	10	–	–
Time to recovery of C3 (days)	–	25	28	7	–	–	33	–
Relapse	–	Yes, 2 years later	No	No	No	No	Yes, 1 year later	No

CKRT: continuous kidney replacement therapy; MPP: methylprednisolone pulse therapy; MMF：mycophenolate mofetil; CTX：cyclophosphamide; CR: complete remission; PR: partial remission.

### Prognosis

The follow-up period was 12–114 months (median 65 months, 57–72 months). Four of 8 patients achieved complete remission, 3 achieved partial remission, and 1 died (Table 3). During the recovery period, the levels of hemoglobin, platelets, LDH, reticulocyte ratio, serum creatinine and urea nitrogen completely returned to normal in 6 patients after 13–41 days of treatment. However, mild anemia was still present in case 8 after 12 months of therapy. The level of complement 3 returned to normal in four patients after 7–33 days of treatment, with 1 patient (case 5) still showing a reduced level. Proteinuria completely disappeared in 4 patients after 3-20 months of follow-up, with occasional reappearance in 2 patients (case 3, case 7) and a partial decrease in 1 patient (case 8).

Relapse occurred in two patients (cases 2 and 7). Case 2 was discharged after partial improvement because of the unwillingness of his parents to continue treatment, and he irregularly received treatment with Chinese medicine. Relapse occurred after 2 years, and the boy received treatment in our hospital again. MMF was adopted to maintain remission after the disease was under control. No recurrence occurred after follow-up during the following 90 months. Case 7 relapsed at 5 months after steroid withdrawal, and the total time of complete remission was 14 months. She received plasma exchange, MPP therapy and later, MMF. No recurrence occurred within the following 42 months.

## Discussion

The CFH family is composed of seven distinct proteins. They include CFH, factor H-like protein 1 (FHL-1), a splice derivative of the CFH gene, and five CFH-related (CFHR) proteins. Anti-CFH Ab-associated HUS is also called autoimmune HUS (AI-HUS), which accounts for 6–56% of aHUS cases [7,8]. Approximately 85–90% of children with anti-CFH Ab-associated HUS have homozygous deletion mutations of CFHR1-CFHR3 [3]. Early diagnosis of anti-CFH Ab-associated HUS can help to improve the prognosis of children. Here, we retrospectively explored the clinical characteristics, treatment protocol and prognosis of children with anti-CFH Ab-associated HUS.

Anti-CFH Ab-associated aHUS predominantly presents in childhood [9]. In this study, the age of onset of children with anti-CFH Ab-associated HUS was 5.83–13.5 years (median 6.67 years, 6.02–9.03 years). Brocklebank et al. reported that the median age at presentation was 8 years (*N* = 17, range, 1 ∼ 15 years) in their anti-CFH Ab-associated aHUS patient population [10]. The average age is significantly higher in anti-CFH Ab-associated HUS than in other types of HUS. Loirat et al. reported that the onset age of children with *Streptococcus pneumoniae*–HUS (SP-HUS) is less than 2 years old, 70% of children with Shiga toxin-producing *Escherichia coli*-HUS (STEC-HUS) have an onset age of less than 3 years old, and 50% of children with cobalamin C deficiency-related HUS (CblC-HUS) have an onset age of less than 0.1 years old. The onset age of HUS children with CFH and CFI mutations is mainly 2 years old and under. Children with diacylglycerol kinase ε-HUS (DGKE-HUS) have an onset age of less than 1 year old [4,11]. Regarding the sex distribution, previous articles showed that children with anti-CFH Ab-associated HUS equally effects males and females in childhood [5]. In this study, the male:female ratio was 1.67:1, and male predominance was observed, similar to the 11:6 ratio (*N* = 17) in the report by Brocklebank et al. [10]. More research is needed to explore the sex distribution.

The main seasons of onset in this study were summer (from May to June) and winter (from November to December). However, Puraswani M et al. reported that the peak incidence of onset for anti-CFH Ab-associated HUS in children occurs from December to April [12]. Shawky S et al. found that the disease onset of AI-HUS is mainly in March and April, with significantly higher rates in school-aged males [13]. In this study, prodromal infection symptoms existed in 87.5% (7/8) of patients, of whom 75% (6/8) had digestive symptoms such as vomiting and abdominal pain, 62.5% (5/8) had symptoms of upper respiratory tract infection such as fever and cough, and 50% (4/8) had coinfection of the respiratory system and digestive system. Reports from European countries, America, Australia, Japan, South Korea and other countries showed that the vast majority of patients with anti-CFH Ab-associated HUS had digestive symptoms during the prodromal period [5,10,14]. In contrast, studies from Indian countries suggested that fever or respiratory infections were are more common, accounting for 55% of cases [12]. These findings suggest a high prevalence of prodromal infections with common pathogens in patients with anti-FH [15] and are in favor of a “two-hit” model according to which where autoimmunity toward CFH could evolve due to infections in genetically predisposed subjects [16]. The high prevalence of the disease in school-aged children together with a predilection for cold weather and associated prodromal symptoms reflects a possible infectious trigger [12].

In this study, all 8 patients presented with severe hemolytic anemia, thrombocytopenia, acute kidney injury, hematuria and gross proteinuria. Extrarenal symptoms such as pancreatitis, pulmonary hemorrhage, gastrointestinal hemorrhage, and hypertension encephalopathy were also observed in these patients. Serum positivity for anti-CFH Ab was present in these 8 children, and 6 patients showed reduced serum CFH levels. Reduced serum C3 levels were shown in 8 patients. Aditi Sinha reported complement C3 levels lower than 70 mg/dl in 62.0% of aHUS children [17]. These results suggest abnormalities of the alternative complement pathway in the pathogenesis of anti-CFH Ab-associated HUS. CFH is a central regulator of the alternative pathway C3 convertase, both in the fluid phase and on cell surfaces. Nozal and Lopez-Trascasa reported that anti-CFH antibodies in aHUS can recognize the C-terminus end of factor H, prevent factor H from binding to endothelial cells, and help in the regulation of the alternative pathway [18].

Kidney pathological changes in children with aHUS may help predict patient prognosis. Morphological features of HUS have been traditionally divided into early changes and late changes on light microscopic kidney evaluation [19,20]. Early changes were found within 2 months of onset, such as fibrin thrombi in capillary lumens, endothelial swelling, bloodless glomeruli, mesangiolysis, fragmented erythrocytes in the mesangium and subendothelial area, glomerular capillary tuft collapse and mucoid intimal thickening. Late changes include duplication of the glomerular basement membrane, mesangiolysis, arterial intimal fibrosis, organization of luminal thrombi, glomerulosclerosis and interstitial fibrosis, all of which occur 2 months after the first clinical presentation. The presence of vascular thrombosis, cortical necrosis, and glomerular sclerosis in the histopathological evaluation correlated with the development of CKD [21]. Three of 8 patients underwent kidney biopsy in this clinical study. Under a light microscope, mild to moderate segmental proliferation of the mesangial region and shrinkage of the basement membrane were observed. Glomerular capillary tuft collapse was shown in 1 case (case 4), and the interval from the onset to kidney biopsy was 45 days. Segmental glomerulosclerosis was observed in 1 or 2 locations in 2 patients (cases 3 and 6), and kidney biopsy was performed 50 and 60 days after onset, respectively. However, cases 3, 4, and 6 did not progress to CKD after 70, 57, and 72 months of follow-up, respectively.

Homozygous loss of the CFHR1 gene [loss2(EXON:2-6)] and heterozygous mutation of the CHFR5 gene (c.669del A) were found in two patients in this study. Previous work has also shown a correlation between the occurrence of anti-CFH aHUS and homozygous deletions in CFHR1/CFHR3 [22]. CFHR1 deficiency is strongly associated with anti-H antibody production. Hofer et al. reported that 82% of CFHR1-deficient aHUS patients had anti-H antibodies [23]. CFHR1 deletion may increase aHUS damage through an antibody-independent pathway. Moreover, CFHR1 has been reported to inhibit C5 invertase activity and the formation of complement terminal complexes [24]. CFHR5 is the only CFHR protein that possesses CFI-dependent cofactor activity, leading to the inactivation of C3b. CFHR5 was also found to inhibit the activity of fluid phase C3 convertase. CFHR5 genetic alterations may play a secondary role in the pathogenesis of aHUS [25].

Eculizumab is a recombinant, fully humanized hybrid IgG2/IgG4 monoclonal antibody directly that directly blocking blocks human complement component C5. The drug was first used in aHUS cases in 2009, and it has been used as a successful treatment for both adult and pediatric aHUS patients [26]. Before the era of eculizumab, anti-CFH Ab-associated HUS patients were recommended for treatment with plasma exchange combined with immunosuppressive therapy [27], but the start time of plasma exchange, and the selection and course of immunosuppressive agents are still controversial. Eculizumab had not been approved in mainland China and many other areas of the world until now. All 8 patients in our study received treatment with plasma exchange combined with MPP in the acute phase. Seven patients achieved complete or partial recovery. Only one patient (Case 1) died of pulmonary hemorrhage on the 9th day. Therefore, plasma exchange combined with MPP in the acute phase can reduce mortality and improve kidney survival. Among the 8 patients in our study, 6 were administered immunosuppressive agents for persistent proteinuria after the acute phase. Three patients received CTX pulse therapy, and the other 3 patients were treated with MMF within six months after the acute phase. Two patients received MMF after CTX therapy for persistent proteinuria and relapse after 14 months. No recurrence occurred in the 2 patients with relapse after MMF treatment. Therefore, long-term immunosuppressive therapy in the maintenance phase can reduce the recurrence rate. It is recommended that immunosuppressive therapy should be maintained for at least 2 years or continue for 1 to 2 years after the disease is completely relieved [28]. If eculizumab is not available, plasma exchange and immunosuppressive therapy are appropriate. However, the best immunosuppressive is undetermined.

In summary, children with anti-CFH Ab-associated HUS were mainly school-aged and predominantly male, with onset times of summer and winter. Digestive and upper respiratory tract infections were common prodromal infections. CFHR1 and CHFR5 mutations were related to the pathogenesis of aHUS. Plasma exchange combined with MPP therapy in the acute phase and CTX or MMF treatment for maintenance can be utilized in children with anti-CFH Ab-associated HUS if eculizumab is not available.

## Data Availability

The data used and/or analyzed during the current study are available from the corresponding author upon reasonable request.
